# Trends in Intake and Outcome Data From U.S. Animal Shelters From 2016 to 2020

**DOI:** 10.3389/fvets.2022.863990

**Published:** 2022-06-14

**Authors:** Jeffrey R. Rodriguez, Jon Davis, Samantha Hill, Peter J. Wolf, Sloane M. Hawes, Kevin N. Morris

**Affiliations:** ^1^Institute for Human-Animal Connection, Graduate School of Social Work, University of Denver, Denver, CO, United States; ^2^Best Friends Animal Society, Kanab, UT, United States

**Keywords:** animal shelters, intake, live outcomes, euthanasia, trend analysis, animal relinquishment, shelter statistics, COVID-19

## Abstract

In this retrospective, exploratory study, intake and outcome data were compiled from 1,373 U.S. animal shelters for which such data were reported consistently across a five-year study period (2016–2020). Linear regression analysis was used to examine the five-year trends and the impacts of the first year of the COVID-19 pandemic (2020) on the overall trends in intake and outcomes in U.S. animal shelters. The results of the analysis reveal that total intake and euthanasia for both dogs and cats significantly decreased over the study period. The adoption, return-to-owner, return-to-field, and transfer (for cats) categories as a percentage of intake all showed significant increases. Live release rates as a function of total intakes and total outcomes for both dogs and cats showed significant increases over the study period. The findings from this study address a critical gap in the field by summarizing emerging trends at the national level in how cats and dogs are being served in U.S. animal shelters.

## Introduction

The efforts to standardize data collection on sources of intake and outcomes for animals in the care of U.S. animal shelter and rescue organizations have been underway since the 1980s. However, relatively little research to date has assessed the overall trends in intake and outcomes nationally. This gap in data collection and comprehensive program evaluation is particularly notable given the growing recognition of the importance of pet keeping on individual and community health and the increasing number of essential pet support services these organizations offer to their communities.

When animal sheltering began in the United States, as early as 1866 with the founding of the American Society for the Prevention of Cruelty to Animals in New York City, animal control efforts were concentrated on removing dogs and cats from city streets to reduce the threat of rabies (1–3). Although removing “strays” would remain common practice for decades to come, it has been suggested that the related issue of “pet overpopulation” received relatively little attention before the 1940s (4). With the 1950s came the first public education campaigns on the subject, followed by spay–neuter campaigns aimed at addressing the issue during the 1960s (4). It was not until the early 1970s, when publications began to draw attention to the increasing number of stray and unwanted dogs and cats in animal shelters (5) that the number of low-cost spay–neuter clinics began to rapidly increase (4). Along with these developments came a growing interest in basic shelter intake and outcome data (e.g., the number of animals admitted, the number euthanized). Intake estimates from the American Humane Association's Animal Shelter Reporting Study, 1985–1988, illustrate just how little reliable data were available at the time. This report estimated, for example, that anywhere between 16.9 and 28.1 million dogs, along with 10.7–17.8 million cats, entered U.S. animal shelters during 1985. Of these, an estimated 9.9–16.6 million dogs and 7.8–12.9 million cats were euthanized (4).

Recognizing that such uncertainty made it difficult, if not impossible, to measure improvements in a particular program's effectiveness—and in the animal sheltering system's capacity to support the community's animals, more generally—researchers called for a more careful accounting of shelter data. In a 1992 editorial, Rowan (6) referred to the lack of accurate data describing the number of animals entering and exiting U.S. shelters as “a statistical black hole,” pointing out that even the number of operating animal shelters was a matter of considerable uncertainty at the time. It was, therefore, “hardly surprising that national estimates of animals euthanized in shelters vary by a factor of two to three” (6).

Although many of the larger animal shelters were beginning to keep “comprehensive statistics on the number of animals handled and their disposition (euthanasia, adoption, and redemption),” the practice was not universal (7). In addition, “there [was] no standard format for keeping statistical information” at the time (8). In 1993, the National Council on Pet Population Study and Policy was established in part “to gather and analyze reliable data that further characterize the number, origin, and disposition of companion animals (dogs and cats) in the United States” (9). Despite the Council's success in compiling data from an estimated 22–23% of shelters in the country, such efforts were hampered “by (1) shelter suspicion about how data would be used if reported publicly, (2) the birth and disappearance of organizations (e.g., rescue groups), (3) changes in the names and locations of shelters, and (4) the lack of a standard definition of shelter” (9).

As recently as 2008, Scarlett (9) lamented that, although

“progress has been made toward eliminating Rowan's ‘statistical black hole'… basic data still elude us, including: the actual number of animal shelters in the country, national shelter estimates of impoundments and dispositions (euthanasia, adoption, redemption), and the effectiveness of programs (e.g., spay/neuter, adoption counseling) in reducing euthanasia.”

An important step in satisfying the need for “basic data” was addressed in 2004 with the adoption of the Asilomar Accords by industry leaders who agreed to a series of definitions that would “provide a standard way to categorize the dogs and cats who comprise the shelter population of the various organizations each year” (10). In 2011, a coalition of animal welfare organizations created the National Database, the management of which would be overseen by an independent nonprofit, Shelter Animals Count (SAC) (11). The organization's Basic Animal Data Matrix, a simple data collection tool, was designed to “facilitate the roll-up or merging of data at the local, regional, or national level by providing a common framework” (12). As of March 2021, SAC has compiled data from 2,046 animal shelters and rescue groups across the U.S. (13).

To build on the efforts of SAC, Best Friends Animal Society (BFAS) began compiling shelter data in 2016, an effort that first necessitated the identification of thousands of organizations across the country considered to be animal shelters (see definition below). Within the BFAS dataset, shelter metrics from SAC were combined with those shared directly with BFAS, as well as those obtained from other sources (e.g., public records) (14). The aim of this study was to use the data collected by SAC and BFAS to measure the trends in both intake and outcome data from 2016 to 2020 across two scales (actual number per year and percentage of total intake per year). These retrospective exploratory analyses of the data identified the emerging trends in the overall capacity of the animal sheltering system to serve animals in communities across the U.S.

## Materials and Methods

### Data Compilation

Intake and outcome data were obtained from SAC and BFAS to generate a nationally representative sample of animal sheltering organizations that reported intake and outcome data consistently over the study period of 2016–2020. Since all data were publicly available or obtained from the organizations with permission to use for research and evaluation purposes, no human subject protection oversight or other forms of ethical approvals were required. For the purposes of this study, a *shelter* was defined as any organization housing animals in a facility, not located in a residence, that is open to the public at least 2 days each week, including municipal shelters (with more than 24 animals admitted annually), private nonprofit shelters with or without a government contract (with more than 99 animals admitted annually), and rescue groups with government contracts. Sources for shelter data that were obtained from SAC and BFAS included voluntary data submissions (including data submitted to SAC or obtained directly from BFAS “network partners” which includes organizations with which BFAS has ongoing working relationships) and other publicly available sources of data, such as organization or government websites. Within our sample population, seven organizations (0.5%) had multiple locations. One organization has four locations, one has three locations, and five organizations have two locations. These organizations that operate multiple facilities may have reported their data in aggregate or broken down by location.

The number of dogs and cats taken into the shelters and the outcomes for those animals were collected according to the industry standards established through the Shelter Animals Count Basic Animal Data Matrix (12). The categories for intake included stray or at-large: animals that were stated to be unowned or free-roaming; owner relinquished: animals that are admitted by their owner, including adoption returns; owner-intended euthanasia: animals surrendered by their legal owner with the intent of requesting euthanasia; transferred in from another agency: animal admissions from another agency either locally or in a different state or territory for adoption or large-scale intake issues; no reason given: no reason for intake was recorded by the organization; and other: includes all admissions not captured above (e.g., animals born in care).

The categories for outcomes included euthanasia: animals that were euthanized by the facility other than those categorized as owner-intended euthanasia or other non-live outcomes; adoption: animals that were adopted, having permanently left the agency's possession, including barn cat programs resulting in adoption (this does not include animals in foster care or “trial” stays); returned to owner (RTO): stray or owner relinquished animals who are returned to their legal owner; transferred out: animals that were transferred to another facility, either locally or in a different state or territory; returned to field (RTF): animals included in intake, already altered, or altered after intake, and returned to stray capture location to be released (often referred to as shelter-neuter-return or SNR); and other non-live outcome: animals that died in care, were lost in care, or were euthanized as a result of an owner-intended euthanasia request; and other live outcomes: live outcomes not captured in the above (not captured in the outcome subtypes, an example would be the barn cat programs in some shelters).

### Sample Description

**Figure 1:**
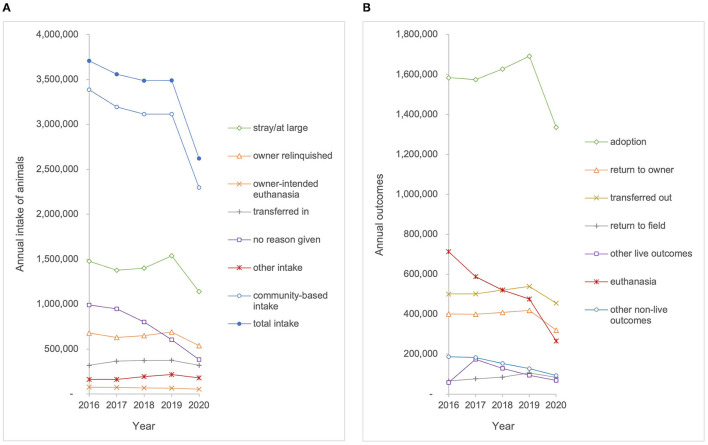
**(A)** Annual intake and **(B)** annual outcomes for animals entering the shelters in the sample population (*n* = 1,373) from 2016 to 2020.

**Figure 2:**
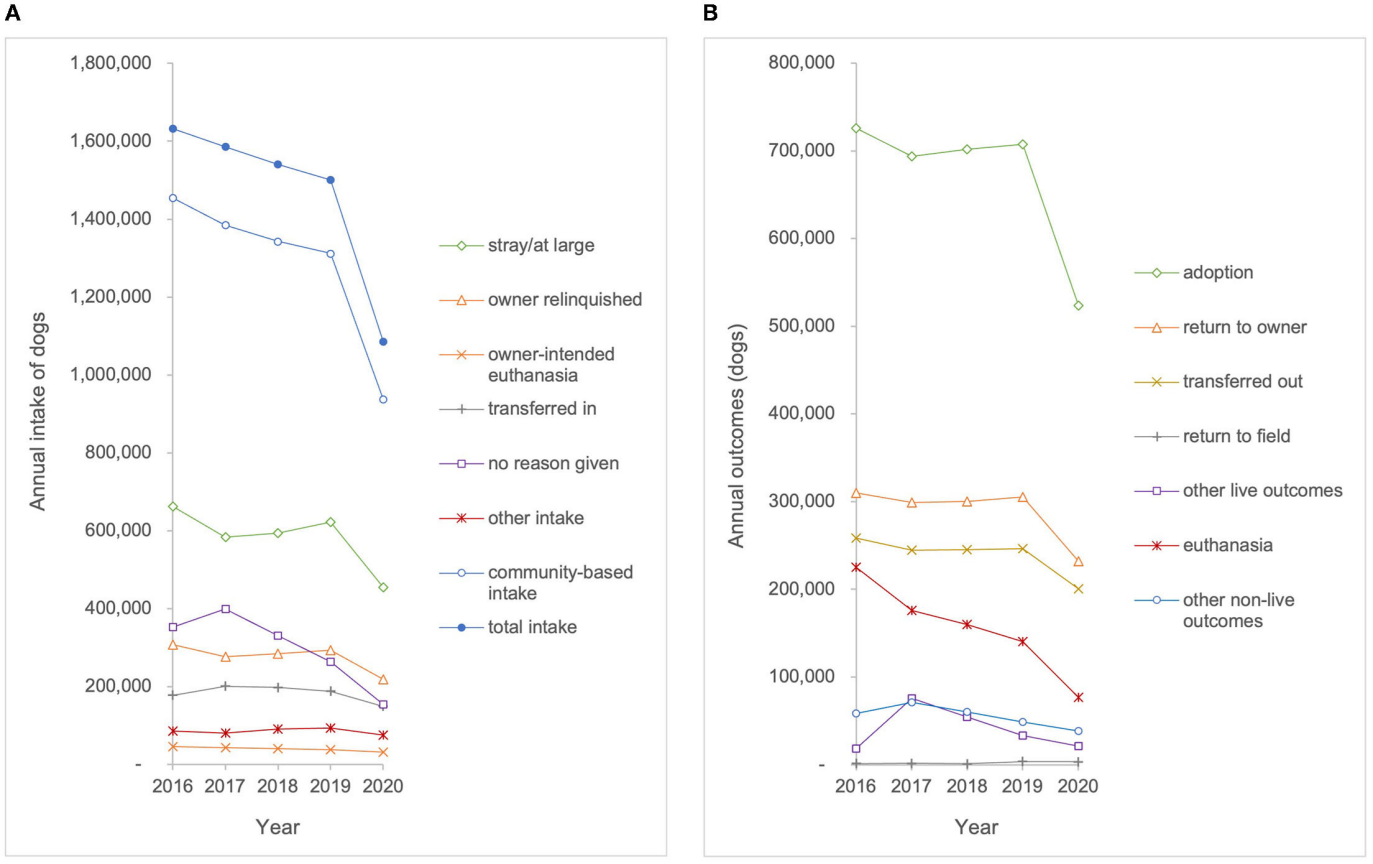
**(A)** Annual intake and **(B)** annual outcomes for dogs entering the shelters in the sample population (*n* = 1,131) from 2016 to 2020.

**Figure 3:**
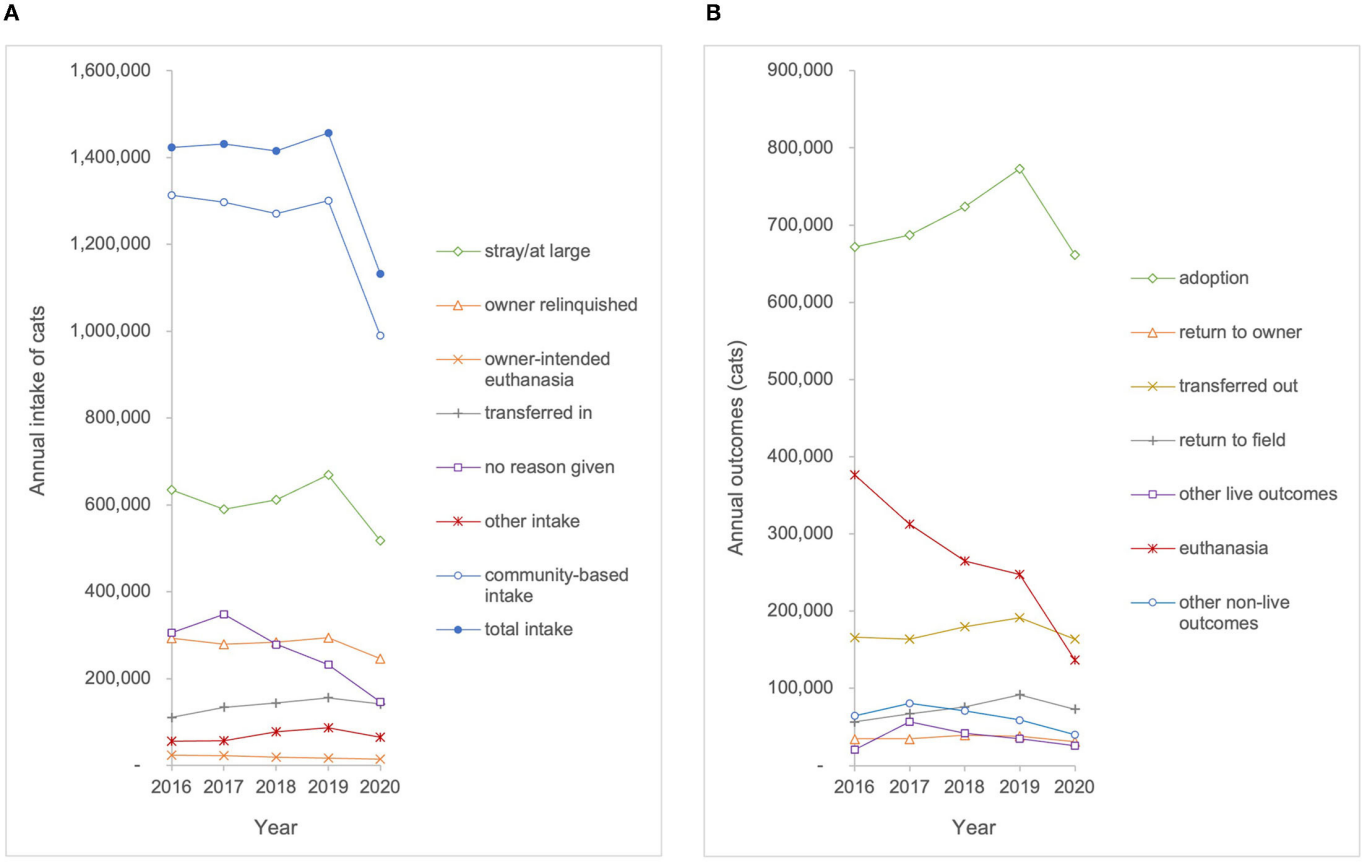
**(A)** Annual intake and **(B)** annual outcomes for cats entering the shelters in the sample population (*n* = 1,101) from 2016 to 2020.

The best estimates identify 4,400 animal shelters across the U.S.; however, data were available from SAC and BFAS for only 3,330 of these organizations (76%) during 2020 and datasets for previous years included fewer shelters (15). For this study, data were compiled for 1,373 total organizations that qualified as a shelter organization and reported data for all 5 years of the study period 2016–2020. The 1,373 organizations include at least one shelter from all 50 states and the District of Columbia accounting for about 31% of the estimated total number of shelters (4,400) in the United States (15). Regionally, the sample is distributed across all eight regions (Southeast *n* = 158; South Central *n* = 160; Pacific *n* = 146; Northeast *n* = 136; Mountain West *n* = 127; Midwest *n* = 140; Mid Atlantic n = 303; Great Plains *n* = 203). This sample of shelter organizations included 676 (49%) government animal service organizations, 388 (28%) shelter organizations without a government contract, 308 (22%) shelters with a government contract, and one (0.0007%) animal rescue with a government contract. Intake and outcome data were aggregated for all animals (both cats and dogs) and analyzed from the 1,373 organizations who reported data over the study period (Figure 1). Species-specific data were only available for a subset of the sample population. Therefore, intake and outcome data were aggregated and analyzed from 1,131 organizations that reported species-specific data on dogs and from 1,101 organizations that reported species-specific data on cats during the study period (2016–2020) (Figures 2, 3). Transfers of animals between multiple shelters may have resulted in some animals being represented in intake data more than once. The maximum proportion of possible intake errors (i.e., individual animals being accounted for twice in intake data), was calculated by dividing the aggregate number of transfers by the aggregate number of intakes for each species (total–25%, dogs–28%, and cats–22%). This calculation assumed that all transfers were between the 1,373 facilities included in the study. To explore the impact of transfers from other agencies on shelter capacity, descriptive statistics were calculated for community-based intake (e.g., stray, owner surrender, and other), often referred to as ‘net intake' in the animal welfare field. This allowed assessment of differences in the trends observed for animals that were admitted to a shelter as a result of community needs (e.g., a lack of access to resources) rather than to facilitate shelter capacity, provide adoptable animals to the community, or optimize resource allocation.

### Statistical Analysis

Trends in the aggregated total, intake subtype, and outcome subtype data (e.g., stray, owner relinquished, adoption, and euthanasia) for the described metrics across the five-year study period were identified by linear regression analysis. Prior to conducting analyses, the assumptions of linear regression were tested for our count and percentage/ratio data by examining normal Q–Q plots, scale location plots, and residual leverage plots. It was determined that the data met the assumptions of linear regression. This exploratory analysis assessed simple increases or decreases over time with the assumption that systemic heteroscedasticity was not an issue and that any changes over the interval were primarily monotonic.

To illustrate the linear nature of the intake and outcome data, Figures 1–3 show raw data of aggregate intakes and outcomes by year for the study period 2016–2020. For linear regression plots, the y-intercept represented the magnitude for each intake category at the beginning of the study period (year 2016) and the slope represented the amount of change per year. No correction for autocorrelation was incorporated into the analyses, although the influence of data from a previous year on the next would have tended to flatten the trends. Slopes with *p*-values ≤ 0.05 were deemed to be significantly different from 0, whereas slopes with *p*-values >0.05 were deemed to have no statistical evidence of a trend during the study period.

To acknowledge the possibility that the COVID-19 pandemic, which began in 2020, may have skewed shelter operations, beginning in 2020, outliers in the dataset were determined using a two-sided Dixon outlier test. An additional linear regression analysis was then completed to explore the trends in the data with any data categories containing significant outliers in 2020 removed for analysis.

No adjustments for the multiplicity of testing were incorporated owing to the exploratory nature of the analyses; thus, the overall type I error could have been greater than the α value (i.e., 0.05) for individual tests. The total change, final value (predicted year 2020 value), and percentage change for the trend line over the study period were calculated for all trends that had slopes significantly different from 0. Total change was calculated as the slope multiplied by the number of years of change that were analyzed (5 years) in the regression analysis. The final value of the trend line was calculated by adding the total change to the y-intercept, and percentage change was calculated as 100 times the total change divided by the y-intercept. For trend lines with slopes not significantly different from 0, the final value was assumed to be the same as the y-intercept.

Data were reported primarily as predicted values from linear regression analyses (not as actual/observed values). This was done because our interest was in examining trends over the entire five-year study period rather than year-to-year changes; reporting only observed values can be misleading since doing so ignores year-to-year variation and may not account for baseline data. Therefore, values for the first year in each analysis (2016) were reported as the predicted y-intercept for the linear regression, and values for subsequent years were calculated from the y-intercept and slope.

## Results

### Trends in Intake

Trends in intake were assessed for all animals, dogs, and cats, based on total intake and intake subtype categories (Table 1). The total intake of all animals entering the 1,373 shelters and rescue organizations included in our sample decreased by 23% from 3,820,931 to 2,925,427 over the study period (*p* < 0.01). Owner-intended euthanasia for all animals decreased by 30% from 79,312 to 55,711 over the study period (*p* < 0.01). Owner-intended euthanasia for dogs decreased 28% from 46,651 to 33,561 (*p* < 0.01), and owner-intended euthanasia for cats decreased from 23,985 to 14,726, a decrease of 39% (*p* < 0.01). No reason given for intake of all animals decreased by 59% from 1,057,237 to 433,754 (*p* < 0.01). For dogs, no reason given at intake decreased by 52% from 406,846 to 193,964 (*p* < 0.05). For cats, no reason given at intake decreased by 50% from 349,118 to 175,190 over the study period *(p* < 0.05). The trendline analysis showed that there was no statistically significant change over the study period for total intake for dogs, total intake for cats, community-based intake, or intake subtypes (stray, owner relinquished, transferred in, and other) for all animals, dogs, or cats.

**Table 1:** Results of linear regression analysis of total intake and outcome data reported by animal shelter organizations in the United States (2016–2020).

**Shelter metric**	**Slope**	*P*-value^*^	**Y-intercept**	**Total change**	**Final value**	**% change**
**Intake**						
***Total intake***	*−223,876*	*<0.001*	*3,820,931*	*−895,504*	*2,925,427*	*−23*
Dogs	−117,771	0.07	1,704,366	NC	1,233,284	NC
Cats	−55,833	0.23	1,483,317	NC	1,259,985	NC
**Total community–based intake**	−226,154	0.07	3,473,481	NC	2,568,864	NC
Dogs	−110,818	0.06	1,507,634	NC	1,064,361	NC
Cats	−64,251	0.15	1,362,842	NC	1,105,838	NC
**Total stray**	−51,342	0.35	1,489,711	NC	1,284,342	NC
Dogs	−37,682	0.13	658,880	NC	508,151	NC
Cats	−15,381	0.47	635,547	NC	574,022	NC
**Total owner relinquish**	−21,860	0.31	681,063	NC	593,622	NC
Dogs	−16,015	0.15	308,526	NC	244,468	NC
Cats	−7,865	0.25	295,354	NC	263,895	NC
***Total owner–intended*** ***euthanasia***	*−5,900*	*0.008*	*79,312*	*−23,602*	*55,711*	*−30*
*Dogs*	*−3,272*	*0.003*	*46,651*	*−13,089*	*33,561*	*−28*
*Cats*	*−2,315*	*0.003*	*23,985*	*−9,259*	*14,726*	*−39*
***Total no reason***	*−155,871*	*0.005*	*1,057,237*	*−623,483*	*433,754*	*−59*
*Dogs*	*−53,221*	*0.046*	*406,846*	*−212,882*	*193,964*	*−52*
*Cats*	*−43,482*	*0.04*	*349,118*	*−173,928*	*175,190*	*−50*
**Total other**	10,091	0.20	164,953	40,364	205,316	NC
Dogs	−629	0.83	86,731	−2,515	84,216	NC
Cats	4,792	0.32	58,838	19,167	78,005	NC
**Total transferred in**	1,007	0.93	348,655	NC	352,682	NC
Dogs	−6,952	0.36	196,733	NC	168,923	NC
Cats	8,418	0.11	120,476	NC	154,147	NC
**Outcomes**						
***Total shelter euthanasia***	*−100,626*	*0.007*	*713,557*	*−402,502*	*311,054*	*−56*
*Dogs*	*−33,256*	*0.006*	*222,104*	*−133,024*	*89,080*	*−60*
*Cats*	*−54,439*	*0.006*	*376,568*	*−217,757*	*158,811*	*−58*
**Total adoption**	−37,782	0.46	1,638,195	−151,128	1,487,067	NC
Dogs	−39,154	0.15	748,958	−156,615	592,343	NC
Cats	6,529	0.71	690,344	26,114	716,458	NC
**Total return to owner**	−14,240	0.32	418,055	−56,959	361,096	NC
Dogs	−15,027	0.16	319,418	−60,107	259,311	NC
Cats	−361	0.78	36,172	−1,442	34,730	NC
**Total return to field**	6,617	0.19	69,967	26,469	96,437	NC
Dogs	622	0.09	852	2,488	3,340	NC
Cats	5,732	0.19	61,441	22,931	84,372	NC
**Total transfer**	−5,395	0.66	514,061	−21,580	492,481	NC
Dogs	−11,292	0.10	261,637	−45,169	216,469	NC
Cats	2,327	0.62	168,230	9,308	177,539	NC
***Total other non–live***	*−24,326*	*0.004*	*197,460*	*−97,302*	*100,158*	*−49*
Dogs	−6,216	0.11	67,770	−24,864	42,906	NC
Cats	−7,080	0.16	77,172	−28,319	48,853	NC
**Total other live outcomes**	−6123	0.74	117,106	−24,492	92,614	NC
Dogs	−3,668	0.70	47,896	−14,674	33,223	NC
Cats	−1,230	0.83	38,512	−4,920	33,592	NC

*Community-based intake categories are indented for clarity. NC, not calculated (i.e., the slope of the trend line was not significantly different from 0)*.

* The p-value was calculated to assess whether the slope of the linear regression line was significantly (P ≤ 0.05) different from 0; these values are denoted with italics. Total change = slope*4(years of study-1). Final value = (total change + y-intercept). Percent change = (total change*100)/y-intercept.

### Trends in Outcomes

Trends in outcomes were assessed for all animals, dogs, and cats, based on total outcomes and outcome subtype categories (Table 1). The analysis identified no statistically significant change over the study period for adoption, return-to-owner, return-to-field, transfer out, or other live outcomes. The euthanasia outcome for all animals in our sample decreased by 56% from 713,557 to 311,054 (*p* < 0.01). Dog euthanasia decreased by 60% from 222,104 to 89,080 (*p* < 0.01). Cat euthanasia decreased by 58% from 376,568 to 158,811 during the study period (*p* < 0.01). Other non-live outcomes decreased by 49% from 197,460 to 100,158 over the study period (*p* < 0.01).

### Trends in Outcomes as a Percentage of Intake

Based on our sample of 1,373 animal shelters, the total number of animals euthanized as a percentage of intake in shelters across the country decreased by 44% from 2016 to 2020 (*p* < 0.01) (Table 2). Dog and cat euthanasia as a percentage of intake also decreased by 45% and 52%, respectively (*p* < 0.01). Adoptions of animals as a percentage of intake increased by 20%, and cat adoptions increased by 24% from 2016 to 2020 (*p* < 0.01). Dog adoptions also increased by 10% percent in the study time frame (*p* < 0.05). Return-to-owner outcomes as a percentage of intakes increased by 13% for all animals entering shelters across the United States (*p* < 0.01). The number of dogs returned to owner also increased by >13% from 2016 to 2020 (*p* < 0.01). Transfers for total animals increased by 27% over the study period (*p* < 0.01). Transfers for cats increased by 26% over the study period (*p* < 0.05). Animals that had other non-live outcomes decreased by 34% (*p* = 0.01). Other non-live outcomes for dogs decreased by 38% (*p* < 0.01), and for cats, the decrease was 48% over the five-year study period (*p* < 0.01). No statistically significant changes were identified for transfers of dogs or for animals that had other live outcomes during the study period.

**Table 2:** Results of linear regression analysis of outcomes as a percentage of intake reported by animal shelter organizations in the United States (2016–2020).

**Shelter metric**	**Slope**	***P*-value**	**Y-Intercept**	**Total change**	**Final value**	**% change**
***Total shelter euthanasia***	*−0.021*	*0.002*	*0.191*	*−0.084*	*0.107*	*−44*
*Dogs*	*−0.015*	*0.004*	*0.134*	*−0.061*	*0.073*	*−45*
*Cats*	*−0.034*	*0.001*	*0.259*	*−0.134*	*0.125*	*−52*
***Total adoption***	*0.021*	*<0.001*	*0.425*	*0.083*	*0.507*	*20*
*Dogs*	*0.011*	*0.02*	*0.437*	*0.043*	*0.480*	*10*
*Cats*	*0.028*	*0.008*	*0.461*	*0.110*	*0.571*	*24*
***Total RTO***	*0.004*	*0.002*	*0.109*	*0.014*	*0.123*	*13*
*Dogs*	*0.006*	*0.02*	*0.186*	*0.025*	*0.211*	*13*
Cats	0.001	0.14	0.024	0.003	NC	NC
***Total transfer***	*0.009*	*0.008*	*0.133*	*0.036*	*0.169*	*27*
Dogs	0.006	0.09	0.151	0.025	NC	NC
*Cats*	*0.007*	*0.02*	*0.112*	*0.029*	*0.141*	*26*
***Total RTF***	*0.004*	*0.002*	*0.018*	*0.015*	*0.033*	*85*
*Dogs*	*0.001*	*0.04*	*0.0003*	*0.002*	*0.003*	*696*
*Cats*	*0.007*	*0.002*	*0.040*	*0.026*	*0.067*	*65*
***Total other non–live***	*−0.004*	*0.01*	*0.053*	*−0.018*	*0.035*	*−34*
*Dogs*	*−0.017*	*<0.001*	*0.174*	*−0.066*	*0.108*	*−38*
*Cats*	*−0.037*	*<0.001*	*0.312*	*−0.149*	*0.163*	*−48*
**Total other live**	−0.0002	0.97	0.031	−0.001	NC	NC
Dogs	−0.001	0.87	0.029	−0.004	NC	NC
Cats	0.00003	0.99	0.026	0.00001	NC	NC

*See Table 1 for key*.

### Live Release Rates

Trend lines indicated that the live release rate (LRR) for dogs as a function of intakes increased by 15% from 2016 to 2020 (*p* < 0.01) (Table 3). The LRR for dogs as a function of outcomes increased by 24% from 2016 to 2020 (*p* < 0.01). For cats, LRR as a function of intakes increased by 12% from 2016 to 2020 (*p* < 0.05). The LRR for cats as a function of outcomes increased by 21% (*p* < 0.01). There was an increase for all animals of 20% for LRR as a function of intakes (*p* < 0.01) and 9% as a function of outcomes (*p* < 0.05).

**Table 3:** Results of linear regression analysis of dog, cat, and total live release rates as a function of total annual intakes and as a function of total outcomes by animal shelter organizations in the United States from 2016 to 2020.

**Shelter metric**	**Slope**	*P*-value*	**Y-intercept**	**Total change**	**Final value**	**% change**
***LRR intakes***	*0.033*	*<0.001*	*0.666*	*0.133*	*0.799*	*20*
*Dogs*	*0.025*	*0.007*	*0.696*	*0.101*	*0.797*	*15*
*Cats*	*0.023*	*0.02*	*0.774*	*0.094*	*0.868*	*12*
***LRR outcomes***	*0.018*	*0.03*	*0.792*	*0.072*	*0.865*	*9*
*Dogs*	*0.036*	*0.008*	*0.597*	*0.143*	*0.740*	*24*
*Cats*	*0.033*	*0.007*	*0.612*	*0.130*	*0.743*	*21*

*See Table 1 for key*.

### Impacts of the COVID-19 Pandemic

The outlier test indicated that there were several categories of shelter operations in 2020 that differed significantly from previous years. The year of 2020 was an outlier in the dataset for the following categories: total intake, total community-based intake, total adoptions, total return to owner, and total transfers out (Table 4). Trends in intake and outcomes with the outlier year of 2020 removed were then assessed. The trend line in total intake for all animals and cats, without the outlier of 2020, did not change from 2016 to 2019. However, total intake for dogs, excluding the outlier of 2020, decreased by 12%. Similarly, the trend line in total community-based intake for all animals and cats, without the outlier of 2020, did not change. However, total community-based intake for dogs, excluding the outlier of 2020, decreased by 13%. The trend lines in adoption, return to owner, and transfer as an outcome, without the outlier of 2020, did not change.

**Table 4:** Results of linear regression analysis of intake and outcome subtypes (2016–2019), with intake and outcome subtypes in which 2020 was an outlier.

**Shelter metric**	**Slope**	*P*-value^*^	**Y-intercept**	**Total change**	**Final value**	**% change**
**Intake**						
**Total intake**	−72,503	0.09	3,669,558	NC	3,379,546	NC
*Dogs*	*−91,037*	0.006^*^	*3,074,449*	*−364,148*	*2,710,301*	*−12*
Cats	8,166	0.41	1,419,318	NC	1,451,982	NC
**Total community-based intake**	−89,928	0.10	3,337,321	NC	2,977,609	NC
*Dogs*	*−46,931*	0.02^*^	*1,443,746*	*−187,724*	*1,256,022*	*−13*
Cats	−6,434	0.53	1,305,025	NC	1,279,289	NC
**Outcomes**						
**Adoption**						
Dogs	−4,746	0.55	714,550	NC	695,566	NC
**Total return to owner**	6,439	0.08	397,376	NC	423,132	NC
Dogs	−1,357	0.66	319,418	NC	313,990	NC
**Total transfer**						
Dogs	−3,475	0.31	253,821	NC	239,921	NC

*Community-based intake categories are indented for clarity. NC, not calculated (i.e., the slope of the trend line was not significantly different from 0)*.

* The p-value was calculated to assess whether the slope of the linear regression line was significantly (P ≤ 0.05) different from 0; these values are denoted with italics. Total change, slope*4(years of study-1). Final value, (total change + y-intercept). Percent change, (total change*100)/y-intercept.

## Discussion

This study included a sample of 1,373 animal shelter organizations across the U.S. While this dataset represents the most representative and accurate estimate of shelter intake and outcome on the national basis to date, there is still a need for increased participation by shelter organizations in reporting data to these national repositories. For example, only 2,386 of the 4,400 animal shelters known to exist in the U.S. (44%) self-reported a full year of data to SAC in 2020 and there was a lack of participation from the Midwestern and Southern regions (16). Some states have addressed this issue of lack of participation by legislatively mandating reporting of shelter intake and outcome data as a condition of licensing [see, e.g., (17)].

The overall trends observed in this study indicate that total intake and euthanasia are decreasing for both dogs and cats. Understanding trends in intake across the U.S. is important for assessing the overall capacity and resources of the sheltering system that could be made available to address community-specific needs. Previous studies have utilized geographic information system (GIS) mapping of intake sources for specific communities to inform program development and resource allocation, particularly for communities with high rates of intake (18). By presenting trends from a representative sample of organizations across the U.S., this study provides useful information on how current programs are impacting animal welfare on a national basis.

### Impacts of the COVID-19 Pandemic

Many animal shelter practitioners are eager to examine how the COVID-19 pandemic has impacted the animal shelter system within the U.S. While there was an overall decrease in the trend in total intake from 2016 to 2020, it is notable that an estimated 2,622,682 million dogs and cats entered shelters in 2020, which represents a decrease of 25% from the 3,489,598 million total intakes reported in 2019. This stands in contrast to the modest change documented between 2018 and 2019, when admissions increased by 0.07%. The restricted services imposed by many shelters during the COVID-19 pandemic may have been a key factor in the decreased admissions recorded in this dataset during 2020. For example, in a statement outlining recommendations for animal control operations during the COVID-19 pandemic, the National Animal Care and Control Association emphasized the importance of reducing shelter admissions:

“Animal control agencies should take active measures to reduce non-essential shelter intake. Measures taken should include returning pets in the field instead of impounding them, suspending non-emergency owner surrender intake, and encouraging owners who are ill to keep their pets at home whenever possible” (19).

Many organizations also embraced a “community-supported sheltering” model during the COVID-19 pandemic and created new programs or increased the availability of existing programs that proactively address the most common reasons for shelter intake (e.g., housing insecurity, access to veterinary care, and access to pet food and supplies). Examples of these programs include pet food and supply banks (20), advocating for pet-friendly rental policies (21), shifting animal control operations from a punishment to support model (22), offering co-sheltering options for individuals in crisis (e.g., individuals experiencing homelessness or domestic violence) (23), One Health vaccine clinics (24), and examining how social and economic inequities affect shelter intake (25, 26). There are a number of other emerging program areas that may also be contributing to the measured decreases in total intake throughout the study period, including low- or no-cost spay–neuter services and other preventive veterinary care (27, 28); door-to-door outreach in underserved communities to overcome barriers in access to veterinary care (29), trap-neuter-return (30–32), and return-to-field programs (33, 34).

This study explored whether the first year of the pandemic (2020) functioned as an outlier in the overall trends in animal shelter intake and outcomes over the last 6 years. The results indicate that 2020 was, in fact, an outlier for the following categories of animal shelter intake and outcomes: total intake, community-based intake, adoptions, return to owner, and transfers out. Any differences in trends that have been observed with or without the 2020 data may be initial indicators of the impacts of the COVID-era programs that have been implemented by shelters in recent years. However, it is important to note that one would not typically remove any single year of data from a trends analysis solely based on the results of any one statistical test. Further investigation is recommended once the additional years of data following the pandemic (2021, 2022) are available, to assess the extent to which 2020 has been or will continue to be an outlier in the dataset. Further, future research should aim to measure the long-term sustainability and effectiveness of these programs while also identifying any remaining gaps in pet support services, particularly in historically marginalized communities.

### Best Practices in Animal Shelter Intake and Outcomes

There are a number of emerging best practices in the animal sheltering field that likely inform the observed trends in intake and outcomes over the study period. The significant decreases in the trends of euthanasia for cats and dogs likely represent a focused effort of both local and national organizations to implement best practices both within the shelter organizations and in the surrounding community. Across the 1,373 animal shelters examined in this study, there were an estimated 265,578 dogs and cats euthanized in 2020, which represents a decrease of 44% from the 475,489 euthanasia outcomes reported in 2019. This stands in contrast to the modest change documented between 2018 and 2019, when euthanasia decreased by 9%. The literature indicates that these best practices for reducing shelter euthanasia include providing specialized medical and behavior care for animals in shelter care, reducing barriers to adoption (e.g., eliminating adoption fees), engaging in triage and appointment-based admissions, and increasing stakeholder engagement in shelter services (e.g., foster caregiving, partnerships with local private practice veterinarians) (35–39). Future research should continue to assess which populations of animals are most likely to be euthanized and continue to evaluate which programs are most effective at reducing non-live outcomes in animal shelters.

This study also observed statistically significant decreases in intake by owner-intended euthanasia and for the “no reason given” category. There has been limited examination of the issue of owner-intended euthanasia, so ongoing assessment of this trend should be studied in future research (40). The “no reason given” category represented a large percentage (22%) of the data available by intake subtype. The significant decrease in this subtype likely indicates a promising improvement in organizations' ability to collect more detailed data. Understanding the factors contributing to this increase in reporting of specific intake subtype data is important to advance the national efforts to compile data from a greater percentage of organizations across the United States.

Although the decreases observed in RTO rates for both dogs and cats were not statistically significant, the considerable differences in RTO as a percentage of intake by species are worth highlighting. While RTO as a percentage of intake increased over the study period by 13% for both all animals and dogs, there was no significant increase, there was no significant increase in RTO as a percentage of intake for cats. By contrast, RTF as a percentage of intake significantly increased over the study period by 65% for cats and 696% for dogs. It is worth nothing that the RTF data for dogs are likely erroneous, almost certainly representing pet dogs returned directly to their owners “in the field” by enforcement staff without bringing the dogs to the shelter. The observed increases in canine RTO and feline RTF rates, each as a percentage of intake, may be a reflection of more organizations returning lost animals to the community where they were found, rather than keeping them in shelter care to be reclaimed through the traditional RTO process. This innovation in lost/found programs for both cats and dogs was implemented within the shift toward community-support sheltering models and effort to reduce shelter intake during the COVID-19 pandemic and was further justified by previous studies that documented RTO rates of 7% or less for cats (8, 41, 42), compared to 15–35% for dogs (8, 41, 43–45). Future research is still needed on the best practices for continuing to increase live outcomes for lost/found animals and community cats and dogs.

While not statistically significant, the decreasing trends in community-based intake should be monitored on a national basis as a promising indicator of how a collaborative animal welfare system, growing emphasis on surrender prevention, and increasing access to pet support services might be making a positive impact on animal welfare outcomes. Transfers are another important strategy for optimizing the shelter system's capacity on the local, regional, and national level that should be monitored in future research. Over the study period, transfers as a percentage of intake increased by 27% for all animals and by 26% for cats, with no change for dogs. Transfers can help facilitate live outcomes for animals, particularly when the organization receiving the transfer has a higher degree of specialization in addressing the medical or behavioral challenge of the animal or has access to a larger population of potential adopters. Future research should monitor efforts to standardize health and safety protocols for transfer partnerships, including best practices such as mandatory quarantine or medical treatment prior to or post-transport. Further, transfers across state lines should be studied at the state or regional level to understand the extent to which this source of intake may impact the community's risk of disease (46–49).

A number of studies have documented trends in animal shelter intake and outcomes on the individual organization or state level (44, 45, 50–53). This study addresses a critical gap in the field to summarize emerging trends in how cats and dogs are being served in animal shelters at the broader national level within the U.S. (54–56). The findings illustrate a comprehensive picture of the changing dynamics of animal shelter intake and outcomes for cats and dogs at the national level that likely impacts the trends observed at the state and regional levels (50, 51). By breaking down the results by intake and outcome type, these data provide insights into pet support services needs across the U.S. and the overall capacity of the national sheltering system to meet those needs. These findings can be used to inform pet support service program development and overall resource allocation in the animal welfare field.

### Limitations

The findings of this study have several limitations. The methods used in recording, compiling, and analyzing data from a national sample of sheltering organizations are not without their shortcomings. One potential limitation is that all shelter data included in the study are self-reported by each organization and assumed to be as accurate as can be reasonably expected. The best estimates identify 4,400 animal shelters across the United States (15). The sample of organizations included in this study (*n* = 1,373) consisted of those organizations who voluntarily submitted data to SAC or BFAS, or otherwise had data publicly available on their websites. In contrast, more than 3,300 animal shelters are included in the 2020 dataset. Future studies examining longitudinal trends will benefit from the larger sample sizes available for more recent years. The relatively low proportion of organizations with publicly available data relative to the number of known organizations represents a potential limitation for this study, while also representing an important future direction for research in this field. It is also worth mentioning that the organizations that report to BFAS through the SAC coalition may have unique characteristics compared to organization that do not report to SAC, which should be considered when interpreting trends using data from this source. Shelters that report their data on this publicly available platform are likely to have higher live release rates, a larger number of animals served annually, and so forth, with leadership who are committed to values around community engagement and transparency of data collection. Organizations that do not report to SAC likely have limited resources available to them (e.g., access to data collection software, dedicated staff time) to report these data on an annual basis. Further limitations of the sample include that Best Friends Network partners are over-represented in the sample of consistently reporting organizations over the study period of 2016–2020 (52% of the sample, compared to 35% of the 4,400 shelters identified). Network partners are organizations with which BFAS has a working relationship, the benefits of which include access to training, various resources, and grant funding. These organizations could have had greater access to information on best practices for decreasing intake and euthanasia; therefore, the efforts to increase representation of organizations with more limited funding or support from national organizations is an important effort for assessing the ongoing needs and challenges in the field. Another limitation of note is that it is likely that there were several pandemic-related factors that are outside the control of animal sheltering organizations (e.g., stay-at-home orders, increases in mental health concerns, and economic barriers due to unemployment or underemployment) that contributed to the trends that were observed in the 2020 timepoint of the dataset. Due to the exploratory nature of this study and the uncertain nature of the post-COVID-19 reality in animal sheltering organizations, these findings should be used to assist in hypothesis generation for future studies rather than drawing definitive conclusions about the trends in national level shelter metrics. Finally, there are likely a number of key factors (e.g., regional heterogeneity, species, facility-type, and total number of animals served) informing the observed trends and assessing the influence of these organizational characteristics on the trends in both intake and outcomes.

## Data Availability Statement

The datasets presented in this article are not readily available. Requests to access the datasets should be directed to: kevin.morris@du.edu.

## Author Contributions

PW, JR, SMH, and KM conceived the study design. JD and SH compiled and analyzed the original dataset. JR performed the statistical analysis. JR, JD, SH, PW, SMH, and KM contributed to the drafting of the manuscript. All authors contributed to the article and approved the submitted version.

## Funding

JR's position is supported by a grant from an anonymous donor to the University of Denver Graduate School of Social Work, and SMH and KM's positions are partially funded by the latter's American Humane Endowed Chair research fund.

## Conflict of Interest

JD, SH, and PW were employed by Best Friends Animal Society, Kanab, UT, United States. The remaining authors declare that the research was conducted in the absence of any commercial or financial relationships that could be construed as a potential conflict of interest.

## Publisher's Note

All claims expressed in this article are solely those of the authors and do not necessarily represent those of their affiliated organizations, or those of the publisher, the editors and the reviewers. Any product that may be evaluated in this article, or claim that may be made by its manufacturer, is not guaranteed or endorsed by the publisher.
